# Determining Potential Feed Value and Silage Quality of Guar Bean (*Cyamopsis Tetragonoloba*) Silages

**DOI:** 10.1515/biol-2019-0038

**Published:** 2019-07-22

**Authors:** Mustafa Olfaz, Unal Kilic, Oguzhan Yavrucu

**Affiliations:** 1 University of Ondokuz Mayis, Faculty of Agriculture, Department of Animal Science, 55139 Samsun-Turkey

**Keywords:** Cluster bean, ecomass, forage, grain, guar, molasses, relative feed value, silage

## Abstract

This study was carried out to determine the effects of some additives on the potential nutritional value and silage quality of guar bean (Cluster bean = *Cyamopsis tetragonoloba*) silages. It was hypothesized that the use of cereal grains, molasses and ecomass will reduce silage fermentation pH by increasing lactic acid production and positively improve CP content and silage quality. Four different silage groups were established; (control, grain (5%), molasses (10%) and ecomass+molasses (10%+5%)). Fresh guar beans were thoroughly mixed with the additives to homogenize, then ensiled and opened after 60 days. The results of this study revealed that guar bean silages could be used as an alternative forage in ruminant feeding because of its higher protein content (13.88%), forage feed value and silage quality. The use of molasses (GSM) and molasses + ecomass (GSEM) as additives has significantly (P ≤0.01) increased the silage quality and feed value compared to the control group. It was observed that GSM and GSEM silage groups had the highest values in terms of lactic acid content. In conclusion, guar silage can be used as an alternative feed for ruminants, but the doses of barley and molasses should be correct and tested in *in vivo* studies.

## 1 Introduction

Fodder plants and crop residues of field, grass and pastures are mainly forage sources for ruminants. However, forage insufficiency is a very important problem in ruminant nutrition. Consequently, some of the shrubs, thorny plants and tree leaves can be used as alternative forages, especially in the wintertime [1, 2, 3]. It is believed that guar bean (Cluster bean = *Cyamopsis tetragonoloba*) will contribute to closing the forage gap [4]. Forage needs of ruminants are provided for by drying or ensiling hay in winter. Guar bean is an annual legume. It is a multipurpose plant and widely cultivated in semi-arid regions such as India and Pakistan. Guar has great potential and because of its low water requirements, it could be an excellent alternative for some forages such as alfalfa [5]. It is an important plant because of the galactomannan gum contained in its structure. Guar bean has the potential to be forage for ruminants because it is rich in leaves and protein, has a high yield and is harvested 3-4 months after planting [6, 7]. However, due to the presence of hydrocyanic acid (HCN) in its fresh state, its consumption by animals is negatively affected. HCN is mostly found in young seeds and rarely found in mature seeds. Some phenolic compounds are present in the guar bean seeds [8] and for this reason, it is recommended to use guar bean silage (GS) in ruminant nutrition. Fresh guar beans has shown very good nutritional composition (CP 3.54%, EE: 0.64%, NFE: 11.86%, NDF: 11.69% for dry matter 25.5%) [4]. It is reported that guar forage is unpalatable and not suitable for grazing but sometimes it can be used to reduce the risk of bloat in grazing ruminants [9,10].

Guar bean silage is being increasingly recognized as an unpalatable high-quality forage resource for ruminants. This study aims to determine the nutrient contents, silage quality and relative feed value of GS, because of the limited literature about guar silage. This study was carried out with the hypothesis that ensiling guar beans with 5% grain, 10% molasses and 5% molasses + %10 ecomass will improve the silage quality and forage nutritional values. It could be difficult to ensile guar beans alone which is a legume forage crop. Thus, it was hypothesized that the use of grains and molasses as additives will reduce the pH in silage fermentation and increase lactic acid production, where the addition of ecomass will positively improve the CP content and the feed value of the silage both organoleptically and chemically.

## 2 Materials and methods

### 2.1 Feeds supply and silage making

Guar bean (Cluster bean = Cyamopsis tetragonoloba) seeds were sown at the Research and demonstration Farm of The Ondokuz Mayis University (1000 m^2^) in the Samsun Province of Turkey (140 m altitude, 41° 21ʹ latitude (N) and 36°15ʹ longitude (E)) and harvested between 16 May 2017 -27 September 2017 at 27.58% DM. In the silage making process, guar beans were harvested 5 cm above the roots. Fresh materials were chopped to about 2-2.5 cm and ensiled into 5 replicate laboratory type PVC silos (2.5 liter, 10 cm diameter and 30 cm length), 5 for each treatment according to Filya [11]. Fresh guar beans were used as silage material and 4 different silage groups have been established (control (GSC=0%), grain (GSG=ground barley 5%), molasses (GSM=10%) and ecomass + molasses (GSEM=10%+5%) added). The molasses contain 50% sugar, the ecomass contains 94% dry matter and 48% crude protein (Ecomass is a feed additive containing 48% CP and 94% DM in dark brown powder form, containing powdered fermentation-derived proteins and complementary carbohydrates (bran and corn gluten), made entirely of vegetable-originated raw materials). Guar bean fresh materials were thoroughly mixed with the additives to homogenize then ensiled. Silos were stored under room temperature (20-25°C) in the laboratory.

### 2.2 Chemical analysis

All the silos were opened after 60 days of fermentation and samples were oven dried at 55˚C for 72 hours. They were then milled in a hammer mill through a 1 mm mill screen size milling machine (Erkaya Laboratory Modal, Turkey) for routine laboratory analysis. Dry matter (DM) was determined by drying samples at 105 ºC for 24 hours. Ash content was determined by burning in a muffle furnace at 550 ºC for 8 hours. Nitrogen (N) content was analyzed using the Kjeldahl method in according to AOAC [12] procedure. Crude protein was calculated as N × 6.25. The ether extracts (EE) content was determined by using an Ankom ^XT15^ analyzer according to the Am-5-04 procedure [13]. The organic matter (OM), nitrogen-free extract (NFE), cellulose (Cel) and hemicellulose (Hcel) contents of the samples were determined through calculation. The analyses of crude fiber (CF), neutral detergent fiber (NDF), acid detergent lignin (ADL) and acid detergent fiber (ADF) contents of the leaves were based on the method of Van Soest et al. [14] using a semi-automatic ANKOM^2000^ fiber analyzer (filter bag technique). The condensed tannin contents of the leaves were determined according to Makkar et al. [15]. All chemical analyses were carried out in triplicate.

### 2.3 Determining pH and VFA, NH_3_-N analysis in the silages

The measured pH (MpH) values of silages were determined at samples obtained from different parts of silages. With this aim, a 25 g silage sample was put in a mixer, 100 ml distilled water added and mixed for 25-30 minutes. Then, the fluid part of the mix was filtered into a beaker via a filter paper and after 15-20 minutes the pH was measured using a digital pH-meter (Hanna Inst. 1332) in three replicates [16].

The required pH (RpH) value in a silage prevents the proliferation of clostridia and enterobacteria. Each silage should have a pH value which is determined according to its DM content. The RpH values were determined by using following formula [17].

Required pH(RpH)=0.00359×DM(g/kg)+3.44

Ammonia nitrogen contents of the silages were determined according to the Kjeldahl method [12]. Lactic acid (LA) and volatile fatty acids (acetic acid (AA), butyric acid (BA) and propionic acid (BA)) contents of the samples were determined using a high-performance liquid chromatography (HPLC) device (Agilent 1100 HPLC; Agilent Technologies) according to Tjardes et al., [18] and Canale et al., [19].

### 2.4 Determining forage and silage qualities

The relative feed value (RFV) of guar bean silages were calculated according to Rohweder et al [20];

Dry matter digestibility (DMD, %)= 88.9-(0.779 x ADF%)

Dry matter intake (DMI, live weight, %)= 120/(NDF%)

Relative feed value (RFV,%)= (DMD x DMI)/1.29

The quality class of the silages were determined by using Flieg’s score (FS= 220 + (2 x dry matter % – 15) –40 x pH) and the total sensory point (according to organoleptic analysis = smell, color and structure of silages by 8 experts) [21].

### 2.5 Statistical Analysis

One-way ANOVA was used (R Statistical Software 2005) in the statistical analyses of the observations [22]. The Shapiro Wilk normality test and Levene Homogeneity of Variances tests were used to test the homogeneity of the variances and it was found that the variances were homogeneous (P=0.05). Duncan’s multiple range test was used for the comparison of mean values. In the study, biological replicates for all the samples were taken as three replicates.

## 3 Results

The effects of additives on the chemical content of guar bean silages as dry matter were given in Table 1.

**Table 1:** Nutrient contents and condensed tannin contents of guar silages (DM %).

	GSC	GSG	GSM	GSEM	Sign.
DM*	25.50 ± 0.42d	27.75 ± 0.67c	31.24 ± 0.15b	33.00 ± 0.34a	0.001
OM	91.33 ± 0.03a	91.53 ± 0.08a	90.72 ± 0.08b	88.77 ± 0.32c	0.001
Ash	8.67 ± 0.03c	8.47 ± 0.08c	9.28 ± 0.08b	11.23 ± 0.32a	0.001
CP	13.88 ± 0.22c	15.00 ± 0.31c	21.64 ± 0.07b	27.59 ± 1.09a	0.001
EE	2.51 ± 0.08b	3.11 ± 0.15ab	2.51 ± 0.33b	3.52 ± 0.33a	0.026
CF	28.45 ± 0.86a	26.36 ± 0.58ab	24.19 ± 0.97bc	23.10 ± 1.00c	0.002
NFE	46.48 ± 0.94a	47.06 ± 0.83a	42.38 ± 1.28b	34.56 ± 0.98c	0.001
NDF	45.87 ± 0.91a	40.73 ± 0.57b	36.54 ± 0.78c	32.28 ± 1.77d	0.001
ADF	32.64 ± 0.43a	28.83 ± 0.30b	26.49 ± 0.35c	21.97 ± 1.18d	0.001
ADL	6.06 ± 0.41	5.26 ± 0.25	5.19 ± 0.20	4.87 ± 0.41	0.113
Hcel	13.23 ± 0.67a	11.90 ± 0.41ab	10.05 ± 0.49c	10.31 ± 0.61bc	0.003
Cel	26.58 ± 0.37a	23.57 ± 0.33b	21.3 ± 0.29c	17.10 ± 1.20d	0.001
CT	0.37±0.03b	0.38±0.01b	0.49±0.02a	0.48±0.02a	0.001

GSC:Guar silage control, GSG: Guar silage grain, GSM: Guar silage molasses, GSEM: Guar silage ecomass+molasses, *DM: Dry matter (as feed), OM: Organic matters, CP: Crude protein, EE: Ether extract, CF: Crude fibre, NFE: Nitrogen free extracts NDF: Neutral detergent fibre, , ADF: Acid detergent fibre ADL: Acid detergent lignin, HCel:Hemicellulose, Cel: Cellulose, CT: Condensed tannin. a,b,c..: Means with different supercripts in the same row are significantly different. Standard error of means (SEM) presented as”±” in the table.

According to the results obtained in this study, the lowest dry matter (DM) content was observed in the control group. In fact, the highest DM content was found in the molasses + ecomass (GSEM) (33.00%) group and it was observed that additives used in the study increased the DM contents of the silages. In the study, the highest CP (27.59%) and ash (11.23%) contents were observed in the GSEM group, and the increase in ash content resulted from the decreased OM content (P ≤0.01). On the other hand, the lowest NFE, NDF, ADF and CF contents were determined in the GSEM group (P ≤0.01). In terms of CT content there were no significant differences between the guar silages; guar silage control (GSC) (0.37%), guar silage grain (GSG) (0.38%), guar silage molasses (GSM) (0.49%) and GSEM (0.48%). However, in the GSC and GSG silage groups, the CT contents were lower than those added with molasses and ecomass (P ≤0.01).

The effects of additives on measured pH, required pH, ammonia nitrogen, volatile fatty acids and lost gasses during the fermentation of silages were given in Table 2.

**Table 2:** The effects of additive on MpH, RpH, NH_3_-N, volatile fatty acids and fermentation gases of silages

	GSC	GSG	GSM	GSEM	Sign.
MpH	5.90 ± 0.13a	5.73 ± 0.05a	4.62 ± 0.07b	4.78 ± 0.03b	0.001
RpH	4.36 ± 0.02d	4.44 ± 0.02c	4.56 ± 0.01b	4.62 ± 0.01a	0.001
NH_3-_N, % Total N	3.47 ± 0.11c	5.84 ± 0.21b	6.69 ± 0.17a	6.18 ± 0.24ab	0.001
LA, %	3.45 ± 0.13c	3.14 ± 0.07c	11.3 ± 0.73b	12.77 ± 0.39a	0.001
AA, %	2.23 ± 0.06ab	1.06 ± 0.12b	3.24 ± 0.72a	2.22 ± 0.11ab	0.021
PA, %	2.67 ± 0.78a	1.55 ± 0.20ab	0.96 ± 0.05bc	0.01 ± 0.00c	0.010
BA, %	5.50 ± 0.63a	5.35 ± 0.29a	0.02 ± 0.01b	-	0.001
GL, %	2.56 ± 0.08	2.75 ± 0.07	2.62 ± 0.21	2.59 ± 0.17	0.799

GSC:Guar silage control, GSG: Guar silage grain, GSM: Guar silage molasses, GSEM: Guar silage ecomass+molasses, MpH: Measured pH value , RpH: Required pH value, LA: lactic acid, AA: Acetic acid, PA: Propionic acid, BA: Butyric acid, GL: Gas loses during fermentation. a,b,c..: Means with different supercripts in the same row are significantly different. Standard error of means (SEM) presented as”±” in the table.

According to this, all the guar bean silages were significantly different from each other (P ≤0.01) in terms of RpH, but there were no differences between GSC and GSG, and between GSM and GSEM in terms of MpH values.

In the study, it was observed that the NH_3_-N content of the silage increased with the use of additives (P ≤0.01) thus lowest ammonia nitrogen (NH_3_-N % total N) were found in the control group (3.47% total N). The highest NH_3_-N contents were in GSM (6.69% total N) and GSEM (6.18% total N) groups (P ≤0.01). The silages were classified according to ammonia nitrogen content [23], while the GSC group had the ‘‘excellent’’ class the others had a ‘‘good’’ class of silage fermentation quality.

High levels of lactic acid (LA) are required to achieve the desired fermentation in silages. Therefore, the highest LA content was determined in the GSEM groups (12.77%), followed by GSM (P ≤0.01). Additionally, GSC and GSG groups were found to be statistically significant in terms of LA (P ≤0.01). In the ensilage process, undesirable butyric acid (BA) contents were not found in the GSEM group, while in the GSM group there was a lower BA than other silages (P ≤0.01). However, in terms of the amount of gas (GL) produced during silage fermentation, it was not an observed additive effect between groups (P= 0.05).

The effects of additives on relative feed values and forage qualities of the guar silages were given in Table 3.

**Table 3:** The effects of additives on relative feed values and forage qualities of the guar silages

Silages	DMD. %	DMI.% LW	RFV	RFV Quality
GSC	63.48 ± 0.34d	2.62 ± 0.05c	128.98 ± 3.23c	Premium
GSG	66.44 ± 0.23c	2.95 ± 0.04bc	151.89 ± 2.58bc	Prime
GSM	68.26 ± 0.27b	3.29 ± 0.07b	174.14 ± 4.19b	Prime
GSEM	71.79 ± 0.92a	3.77 ± 0.22a	210.19 ± 15.14a	Prime
Sign.	**0.001**	**0.001**	**0.001**	

GSC:Guar silage control, GSG: Guar silage grain, GSM: Guar silage molasses, GSEM: Guar silage ecomass+molasses, DMD: Dry matter digestibility, DMI: Dry matter intake, RFV: Relative feed value. a,b,c..: Means with different supercripts in the same column are significantly different. Standard error of means (SEM) presented as”±” in the table. According to the Quality Grading Standard assigned by The Hay Marketing Task Force of the American Forage and Grassland Council, the RFV were assessed as roughages based on prime =151 , 1 (premium) 151-125, 2 (good). 124-103. 3 (fair). 102-87, 4 (poor). 86-75, 5(reject).< 75.

In this study, the GSC group was placed in ‘‘premium’’ but others were ‘‘prime’’ quality class. Therefore, they were placed into a high-quality forage class in terms of RFV. However, the highest DMD, DMI and RFV values were found to be GSEM while the lowest DMD values were found in the GSC (P≤0.01).

Quality classes of the samples were given in Table 4 according to organoleptic and Flieg’s scores.

**Table 4:** Quality classes of guar silages according to organoleptic and Flieg’s scores

Silages	Smell	Structure	Color	Total Point	Organoleptic Score	Flieg’s Point	Flieg’s Score
GSC	9.58	3.20	1.60	14.40	Satisfactory	20.16±5.50c	Fair
GSG	12.20	3.66	1.64	17.50	Good	31.14±2.75b	Fair
GSM	12.80	3.35	1.24	17.40	Good	82.68±2.58a	Excellent
GSEM	13.40	3.30	1.48	18.20	Good	79.72±1.56a	Good
Sign.						**0.001**	

GSC:Guar silage control, GSG: Guar silage grain, GSM: Guar silage molasses, GSEM: Guar silage ecomass+molasses a,b,c..: Means with different superscripts in the same column are significantly different. Standard error of means (SEM) presented as”±” in the table.

In this study, according to the organoleptic tests conducted by 8 experts in this field, silages with additives had higher ‘‘good’’ quality scores than the control group (GSC) in terms of smell, structure and color. According to sensory scoring, the control group of the silages was classified as ‘‘satisfactory’’. For this reason, the use of additives in guar silages have positive effects on organoleptic scoring. According to Flieg’s scoring, the highest quality score ‘‘excellent’’ was observed in the GSM silage groups followed by GSEM which was placed in the ‘‘good’’ quality class. In Flieg’s scoring, there was no significant difference between the GSC and GSG and the effect of barley usage as additive on silage quality was not statistically significant (P ≤0.01).

## 4 Discussion

The NFE content of the GSEM and GSM groups was lower than the control group (GSC), which resulted from the consumption of lactic acid bacteria (LAB) during silage fermentation to meet their energy requirement. The NFE content in the silage material increases the LAB number among microorganisms, thus the pH decreases rapidly, and the silage fermentation occurs in the desired direction. Pancholy and Mali [24] reported that the CP content of silage made from 75% guar beans (25% pearl millet) was 14.57% whereas those silages prepared entirely from guar beans were 11.10%. This value agreed with the result obtained in control group of our study. Alaca and Ozaslan Parlak [25] determined the silage pH, CP, ash and NDF contents as 5.17, 11.93%, 9.02% and 43.70% respectively. Suliman et al. [7] reported the guar silage with a pH value of (4.32), CP (14.60%), EE (2.47), CF (25.82) and NFE (42.16%) contents, in this study similar results were observed in the control (GSC) group. However, the ash content (14.95%) reported by Suliman et al. [7] was higher than the content determined in this study. Sharma et al. [8] determined CP, EE and ash contents for 3 variety of guar bean flours (grain) respectively as 24.55-26.78% DM; 2.70-3.06% DM and 3.59-5.29% DM.

In addition, the pH value found in this study was higher than the values reported by both previous researchers. This variation resulted from soil structure, species difference, fertilizers, and many other factors such as silage material DM, NFE contents, and silage fermentation differences [21]. The condensed tannin (CT) content determined in the guar silage would not adversely affect the ruminants consumption, because it was below the level 5%, which limits its use in animal feeding [3]. This may be due to the fermentation degradation of tannin compounds (Hydrocyanic acid) which may be present in the fresh material. Guar silages may therefore be considered as a source of roughage in the animal feed, which would not create a problem in terms of CT content.

It is known that condensed tannin contents of 5-10% may result in disgust of feeds, decreased digestibility, reduction of forage consumption, live weight gain, absorption, reduced performance and can induce toxic effects [26]. In this study the CT contents of GSC, GSG, GSM and GSEM groups were determined between 0.37-0.49%. It is believed that this will not have an adverse effect on the feed consumption. As a result, it was concluded that guar silages can be utilized as alternative forage for ruminants. Thus, this would have economic benefits and advantages for the closure of the forage feed gaps.

As shown in Table 2, there was a decrease in MpH value in silages with added molasses and ecomass (GSM, GSEM) and the lactic acid bacteria in the fermentation increased. MpH was numerically decreased in the grain added silages, but it was not statistically different from the control (GSC) group. For this reason, the result shows that when cereal grains are used as additives to guar silage, the amount added should be more than 5%. In addition, the MpH value of the GSG group being higher than the RpH value also supports this result. According to this situation, it is possible to say that the level of additives used in both treatments were sufficient, because the RpH and MpH values of GSM and GSEM were close to each other.

Pancholy and Mali [24] ensiled guar bean and millet plants together and established 4 different silage groups consisting of 25%, 50%, 75% and 100% of guar bean respectively. The researchers reported that the pH value of guar bean alone silage was higher than others (4.50), whereas the highest CP content was 75% guar bean-based silages and the lowest LA content (1.62) was the guar bean alone silage. The pH value obtained in this study was higher than those reported by the researchers. In terms of LA content, the results of GSC in this study were higher than those reported by the researchers. However, the decrease in pH and the increase in LA contents of silage when used additives were found similar.

As shown in Table 2, it was observed that GSM and GSEM silage groups were at the desired levels in terms of LA and BA contents and there was no problem related to the additives used in the silage making. This situation also reveals the fact that the amount of grain used in GSG was not sufficient.

In the study, the highest RFV was observed in the GSEM silage group (Table 3) and it was classified as the highest quality class ‘‘Prime’’ with GSG and GSM. In this study, the determination of the control silage group as premium quality shows that the guar bean silages are a higher quality forage and have an important potential in ruminant feeding.

According to Sensory scoring, the addition of grain (barley), molasses and ecomass to guar silage has a positive effect on silage quality. However, according to Flieg’s score, it was determined that grain (barley) added silages (GSG) were similar in quality to control group (GSC), while GSM and GSEM groups were higher quality silages. According to this, in the case of using grains as additive to guar silage, it is recommended to add a dose higher than 5%.

The Flieg’s scores of maize, grass, alfalfa and vetch silages reported by Kilic [27] were significantly higher than those of GSC and GSG groups, but were similar to the Flieg’s quality scores of GSM and GSEM. Therefore, it is possible to say that guar bean silage with the addition of molasses and ecomass can be an alternative to common forage sources.

The guar bean, which has the possibility of being planted as a second crop, is thought to be an excellent roughage source considering the higher yields (2.5 ton / ha) and that it can be harvested in a short time (2.5-4 months). However, ensiling guar bean with millet is known to increase the silage quality [24] and it is recommended to plant guar bean and graminaes together to ensilage.

Due to the lack of enough scientific studies on guar bean silage, the comparison of literature reports in the discussion section could not be made, but GSM and GSEM groups were recommended according to the results of this study. However, when sensory analysis is taken into consideration, reducing the dose of molasses (5%) is recommended. In addition, in future studies, it is recommended to increase the applied dose (above 5%) of cereal grains to the guar silages. Suliman et al. [7] reported that guar forages (fresh hay or silage) improved economic efficiency, daily gain and feed conversion for lambs; and the forages as a partial replacement of concentrates and some (rice) straws up to 40% for lambs rations. Besides, Chiofalo et al [28] reported that guar gum industries depend largely on the utilization of guar seed meal and this product can be considered a valuable feed resource. However, researchers recommended harvesting at 50% pod formation for the guar bean [29].

## 5 Conclusions

According to this study, it was found that the roughage quality and nutrient contents of guar silage are acceptable. The guar silage with molasses+ecomass (GSEM) were higher in lactic acid production, crude protein and dry matter contents. Therefore, molasses and ecomass should be used together in ensiling guar beans to improve silage quality. However, it is recommended that the results obtained from this research should be tested in *in vivo* studies, before these products can be advanced further in ruminant nutrition.

## Abbreviation List

AA: Acetic acid
ADF: Acid detergent fibre
BA: Butyric acid
CF: Crude fibre
CP: Crude protein
CT: Condensed tannin
DM: Dry matter
DMD: Dry matter digestibility
DMI: Dry matter intake
EE: Ether extract
GL: Gas losses during fermentation
GSC: Guar silage control
GSEM: Guar silage ecomass+molasses
GSG: Guar silage grain
GSM: Guar silage molasses
LA: lactic acid
MpH: Measured pH value
NDF: Neutral detergent fibre
NFE: Nitrogen free extracts
PA: Propionic acid
RFV: Relative feed value
RpH: Required pH value
